# The Right to Health: COVID-19 Pandemic and the Opportunity to Transform Mental Health Inequalities in Indonesia

**DOI:** 10.3389/fpubh.2022.844656

**Published:** 2022-03-29

**Authors:** Gina Anindyajati, Diashati Ramadhani Mardiasmo, Laras Sekarasih, Damar Susilaradeya, Bagus Takwin, Dicky C. Pelupessy, Hervita Diatri

**Affiliations:** ^1^Department of Psychiatry, Faculty of Medicine, Universitas Indonesia, Jakarta, Indonesia; ^2^Medical Technology Cluster, Indonesia Medical Education and Research Institute, Faculty of Medicine, Universitas Indonesia, Jakarta, Indonesia; ^3^Faculty of Psychology, Universitas Indonesia, Depok, Indonesia; ^4^Department of Physiology and Biophysics, Faculty of Medicine, Universitas Indonesia, Jakarta, Indonesia

**Keywords:** COVID-19, pandemic, Indonesia, mental health service, psychosocial support

## Abstract

The COVID-19 pandemic has caused major catastrophes worldwide. In Indonesia, the pandemic has caused greater barriers for individuals to access mental health services. This article aims to capture the state of public mental health in Indonesia using data from various national surveys. Four main problems were identified: the increase in depression, loneliness, and distress in the general population, disruption in accessing mental health services, mental health problems among vulnerable populations, and the limited scope of available mental health services and facilities in the community. This article provided practical recommendations for the Indonesian government that focuses on preparing a resilient mental healthcare system for future crises, reducing barriers to access mental health services, and expanding the available resources and programs to ensure equal and sustainable access to mental health services in the community.

## Introduction

As the largest diverse archipelagic country and the fourth most populous country in the world, Indonesia faces unique challenges in controlling the COVID-19 pandemic (1). First, as a low-level resource country in the southeast Asia region, tremendous efforts are required to keep the number of active cases and virus transmission low. Before the pandemic, the physician to population ratio was lower than the internationally recommended ratio. Moreover, the ratio of hospital beds to population remained below WHO standards and trails behind neighboring countries (2). The archipelago also requires connectivity to distribute medical supplies and the COVID-19 vaccines to different parts of Indonesia. The country's geographical position has also put Indonesia at risk for natural disasters, 2,059 natural disasters were recorded between January and September 2020, creating another layer of complexity in managing the COVID-19 pandemic (3). Second, the pandemic has shown to throttle Indonesia's economic growth as Indonesia went from upper-middle income to lower-middle income status and the national unemployment rate has remained at a high 6.49% percent in August 2021 (1, 4). The uncertainty the pandemic poses with the rise and fall of cases, changes in restriction policies as well as the economic stress, not only has introduced risks at the societal level, but also at the individual level.

One of the challenges that seems to be less overt than the physical and financial stress among Indonesians is mental health problems (5, 6). The requirement for physical distancing and limited spatial mobility during the pandemic, while crucial in slowing down the virus transmission, have created some constraints for individuals to maintain their psychological wellbeing. The third phase of COVID-19 vaccination rollout included people with mental illnesses and the Indonesian government even provided door-to-door vaccination services to people who were severely mentally ill however, this was still insufficient in facilitating routine access for their recovery (7, 8). Unfortunately, equal and affordable access to mental health facilities remains one of the issues in many parts of Indonesia, even among individuals residing in the country's major cities.

This perspective is written based on a collaborative work between mental health professionals, academics, and administrators with broad expertise on mental health and public health. The objective of this perspective is to identify priority issues in mental health and provide specific recommendation to anticipate the effect of COVID-19 pandemic toward Indonesia's mental health system.

## Main Issues

This section provides our report on priority issues regarding the burden of mental health problems in Indonesia during COVID-19 pandemic.

### High Proportion of Depression, Distress, and Loneliness

Proportion of depressive symptoms between March and May 2020 during the pandemic reached 35%, 5–6x higher than the current incidence of depression in Indonesia and 1.5x higher than depression rates seen in other (non-pandemic) disasters (9–11). Periods of quarantine and self-isolation causes loneliness and a sense of deprivation which can lead to suicidal thoughts. According to Indonesian Psychiatric Association (May 2020), mental health issues was observed highest among 17–29 years old and >60 years old (12). They observed suicidal thoughts in 49% of respondents who showed depressive symptoms. A year later, numbers were still reportedly high (39.3%) (13). Fluctuations in social restriction policies may also contribute to anxiety and loneliness. A nationwide survey in November 2020 by Universitas Indonesia Big Data Synergy Against COVID-9 Team found 42.4% of participants felt lonelier since the pandemic (14). In Mei 2021, Into the Light Indonesia reported almost double the number (13), 98% of participants experienced loneliness within the past month.

The pandemic has taken a mental toll on HCWs. Moderate-severe burnout syndrome was found in 83% of Indonesian HCWs, 41% had moderate-severe emotional fatigue, 22% had moderate-severe loss of empathy, and 52% had moderate-severe loss of confidence (15). Compared to pre-pandemic, these numbers have doubled (16–18). Data regarding prevalence of burnout among HCWs pre-pandemic is very limited. A survey by KOMPAS (local newspaper) identified financial stress as the highest (57.6%) type of distress caused by the pandemic, most likely due to a surge in unemployment and a decrease in income (14, 19). In addition, sleeping difficulties are common during the pandemic and a high prevalence was observed in COVID-19 patients (57%) (20). The pandemic has limited opportunities to engage in physical activities due to the state-mandated requirement to stay at home. Both sleeping difficulties and lack of physical activity were associated with depression and anxiety (20, 21). Dynamic changes in living circumstances, policy fluctuations, health uncertainties, and financial burden brought on by the pandemic has resulted in high levels of depression, distress, and loneliness.

### Disruption of Mental Health Services

During the COVID-19 pandemic, a series of mental, neurological, and substance (MNS) related services such as psychotherapy, counseling, mental health interventions, suicide prevention programs, and many others have been completely or partially disrupted in 93% of countries worldwide (22). In Indonesia, one of the main issues is difficulty in accessing healthcare facilities for mentally ill people. Data from 2018 showed a shortage of mental health facilities. Of 9,000 primary-care facilities throughout Indonesia, only 40% have operational mental health programs (23). Only 60% of all hospitals have mental health programs and 6 out of 34 provinces in Indonesia do not have a psychiatric hospital (24). At Dr. Cipto Mangunkusumo General Hospital, a national referral tertiary hospital, quota for psychiatry ward and psychiatry outpatient clinic were significantly reduced during COVID-19 surge e.g., in the psychiatry ward, 24 beds were reduced to 2 and in the outpatient clinic, only 20 patients/day were allowed (adult and geriatric patients combined). This was done to maintain physical distance and as a result of resource allocation to COVID-19 unit (beds, nurses, and resident doctors). At primary-care facilities, general practitioners, and community leaders have not been able to conduct home-visits, consequently severely mentally ill patients who relied on these home-visits for routine check-ups and monthly prescriptions, have had their treatments halted.

People with mental illnesses are at a higher risk for transmitting COVID-19 due to numerous factors such as: (1) self-care limitations (poor hygiene and unhealthy lifestyle); (2) co-morbidities e.g., diabetes; (3) densely populated living environment poses social distancing challenges. One study showed that people with depression and schizophrenia were 7 times more likely to be infected with COVID-19 (25). Those with mental disorders were also associated with an increased risk of hospitalization and COVID-19 mortality (26, 27). On top of all this, mental-illness related stigmatization acts as a barrier to mental health as well as healthcare services in general (28, 29). Thus, these disruptions are disastrous as the need for mental health services during the pandemic is higher than ever.

### Increased Mental Health Issues Among the Diverse Vulnerable Population

Sandwich generation refers to a group of people (usually working population) who simultaneously care for their children and aging parents, causing immense emotional distress during the COVID-19 pandemic, thereby rendering them vulnerable to mental health problems. The pandemic increases the risk of domestic conflict, divorce, elderly, and child abuse. Victims of abuse may feel unsafe at home so they desperately opt to “escape” their homes despite the risk of COVID-19 transmission. Children and adolescents are vulnerable to mental health issues as the pandemic has caused significant learning as well as social changes, since it now heavily relies on technology. Marginalized young people are a community of young adults who are usually homeless, LGBTQ+, disabled, and/or HIV positive. They live in such poor conditions and are already prone to mental health issues, therefore the pandemic only exacerbates their problems. More than half of Indonesian marginalized youths from sexual minority groups (intersex, transgender, non-binary, non-heterosexual) were reported have suicidal and self-harm thoughts (13). One Indonesian study found particular individuals were more vulnerable to anxiety, including those younger in age, of the female sex, suspected COVID-19 infection, and lack adequate social support (30). Lastly, within the geriatric population, apart from feelings of loneliness and abandonment, periods of quarantine can also worsen cognitive function (31). Those among the vulnerable population are already susceptible to mental health problems and the pandemic has amplified their susceptibility.

### Limited Scope of Mental Health Services Within the Community

In Indonesia, identification of mental health issues does not reach all varying layers of society due to lack of access to independent mental health assessment. Current mental health assessment utilizes psychological self-assessment online questionnaire which is not equipped with an adequate referral system (http://pdskji.org/home). Moreover, current healthcare services have failed to integrate both mental and physical aspects of health as well as community-based mental health services, contributing to the limited scope of mental health services in Indonesia.

During the COVID-19 pandemic, the dynamic of mental health services has shifted from in person counseling to e-counseling (telemedicine) due to social restrictions. As of May 2021, 68% of people access mental health services through a phone application or via website (13). Although the new norm of voice and video call consultation is deemed acceptable, its practical use is limited. Those who are digitally illiterate, have no stable internet connection and/or a smartphone, are at a disadvantage. Another important issue is telemedicine services not covered by JKN (National Health Insurance), therefore patients may be discouraged from using telemedicine. Other issues include shorter consultation period, lack of physical examination (e.g., examinations to assess anti-psychotic side effects) and troubles with tele-pharmacy (i.e., inter-province prescription writing is prohibited). A survey by Department of Psychiatry, Universitas Indonesia (32) revealed these mental health service changes were perceived as “less convenient” for patients.

Moreover, misperceptions and poor knowledge regarding mental health issues are common among Indonesian. Into the Light Indonesia found 7 out of 10 respondents admitted to not knowing that mental health expenses were covered by BPJS (Healthcare Social Security Agency) and 3 out of 5 respondents did not know there were mental health facilities within their sub-district (13). Additionally, none of the respondent was able to correctly answer questions regarding suicide facts and myths. All of these key points contribute to the limited scope of mental health services in Indonesia.

## Recommendations

In this section we outline recommendations to improve access to mental health services and ensuring its continuity for people who need it the most.

### Preventing a Mental Health Crisis During and After COVID-19 Pandemic

Prevention of mental health crisis is not solely the responsibility of Ministry of Health. Mental health service is bigger than just healthcare, therefore to deal with such concerns, it will need collaboration between COVID-19 taskforce, Ministry of Health, Coordinating Ministry for Economic Affairs, Ministry of Communication and Information Technology, Coordinating Ministry for Human Development and Cultural Affairs, and BPJS (Healthcare Social Security Agency). Based on the problems listed above, it is recommended to:

- Conduct periodic surveillance on the impact of COVID-19 on mental health issues and its effects toward productivity, work performance, economic wellbeing and social security.
- Conduct surveillance on mental health resources within all types of healthcare facilities.
- Provide digital access to those self-isolating both at home or at a healthcare facility (33, 34) so they have access to relevant information and can continue to communicate with family and friends as well as consult with healthcare professionals online.
- Increase the number of primary-care facilities with operational mental health programs.
- Develop a “Psychosocial and Mental Health Support Team” which includes trained personnel and medical professionals that creates and assists with long term support programs (35, 36); inclusive for the general population and HCWs, easily accessible and inter-connected from sub-district to provincial level.

### Ensuring Continued Services to Mentally Ill Patients

Lack of access during the pandemic has caused disruption of mental health services. Continuity of care is especially critical for mentally ill patients as it prevents decompensation and other consequences (37). Therefore, it is essential to ensure that people with mental illness can access mental health services (38). COVID-19 taskforce and Ministry of Health should collaborate to:

- Provide telemedicine and hotline crisis services (39, 40).
- Ensure stable patients receive enough medication for 2–3 months.
- Provide a system in place for patients who were lost to follow up so they still receive medication.
- Ensure availability of medication in accordance with national formulary standard.

As for COVID-19 prevention, it is crucial ensure that each patient with severe mental illnesses, receive the standard of care by providing them with information on COVID-19 health precautions, educating them on the importance of family and community support to prevent COVID-19 infection, and having community leaders and/or HCWs reach out to them directly.

### Providing Psychosocial and Mental Health Support to the Working Population and Other Vulnerable Population

It is important to ensure the provision of psychosocial and mental health support to all during COVID-19 pandemic (41). It is considered particularly important to provide such support for the working population, people living with HIV/AIDS, children and adolescents, elderly, women, and marginalized young people due to their high exposure to stress (26, 42). Collaboration between COVID-19 taskforce, Coordinating Ministry for Economic Affairs, and Coordinating Ministry for Human Development and Cultural Affairs is required in order to:

- Ensure social security networks are active and working effectively.
- Provide psychosocial support to those who struggle to adapt with working from home/online school and evaluate its effect on their mental wellbeing.
- Develop a guidebook that focuses on how to develop better interpersonal, self-regulation, and communication skills to facilitate the challenges of quarantine.

### Expanding the Scope of Mental Health Services Within the Community

Considering the shortage of mental health facilities, unequal distribution of competent resources, failure to integrate both mental and physical aspects of health as well as community-based mental health services, it is necessary to develop strategies to expand the scope of mental health services within the community. Several recommendations include:

- Provide access to integrate both physical and mental health services, which consists of assessment for anxiety and depression. This access should be in accordance with clinical practice guidelines and should utilize professional and competent human resource. This access should also facilitate online and offline referral systems.
- Provide psychological and emotional support that is integrated with COVID-19 health services available for patients, patient family and healthcare professionals.
- Develop a guidebook that provides information on where to seek help for those suffering from mental health issues/symptoms. Ensure guidebook are readily accessible at primary-care facilities and are disseminated to target populations.
- Conduct routine community outreach activities (43), especially to those isolated from technology.

To achieve these objectives, it will be crucial for COVID-19 taskforce, Coordinating Ministry for Human Development and Cultural Affairs, and Ministry of Health to all work together.

## Conclusion

We identify four priority mental health issues, including high proportion of common mental disorders, service disruption, increased risk among the vulnerable population, and limited service within the community. Therefore, we recommend collaboration between multi-sector government bodies involved in the COVID-19 response and beyond to anticipate the effect of COVID-19 pandemic toward Indonesia's mental health system. The aforementioned bodies include but are not limited to healthcare regulators, funders and providers, such as the COVID-19 taskforce, Ministry of Health, Coordinating Ministry for Economic Affairs, Ministry of Communication and Information Technology, Coordinating Ministry for Human Development and Cultural Affairs, and BPJS (Healthcare Social Security Agency). It is important to optimize utilization of established infrastructure in order to prevent mental health crises due to the pandemic. We also suggest the government provide mental health and psychosocial support, emphasizing on the need of working and other vulnerable populations. For mentally ill patients, we must ensure they receive continuous treatment. Furthermore, with the available resources, we should start to integrate mental health services into current health programs in the community to expand its reach. Other pandemic-related issues such as, effectivity, restriction policy challenges, virus mutations, changes in values and culture, are important areas that would be interesting to study for future evidence-based policies.

## Data Availability Statement

The original contributions presented in the study are included in the article/supplementary material, further inquiries can be directed to the corresponding author/s.

## Author Contributions

GA, DM, LS, DS, BT, DP, and HD: conception and design of the article and writing. All authors read and approved the final version of the article.

## Funding

This article received funding from Ministry of Research and Technology/National Research and Innovation Agency of Republic Indonesia (Grant No. 134/FI/P-KCOVID-19.2B3/IX/2020).

## Author Disclaimer

This article was originally published as a policy brief on August 3rd, 2020. Link: https://sinergimahadata.ui.ac.id/policy-brief/. This article was presented to the Coordinating Ministry for Economic Affairs on August 7th, 2020. Link: https://republika.co.id/berita/qfqkrn327/ui-rekomendasikan-4-kebijakan-kesehatan-mental-saat-pandemi.

## Conflict of Interest

The authors declare that the research was conducted in the absence of any commercial or financial relationships that could be construed as a potential conflict of interest.

## Publisher's Note

All claims expressed in this article are solely those of the authors and do not necessarily represent those of their affiliated organizations, or those of the publisher, the editors and the reviewers. Any product that may be evaluated in this article, or claim that may be made by its manufacturer, is not guaranteed or endorsed by the publisher.
